# IGF-1 enhances cell proliferation and survival during early differentiation of mesenchymal stem cells to neural progenitor-like cells

**DOI:** 10.1186/1471-2202-15-91

**Published:** 2014-07-22

**Authors:** Tee Jong Huat, Amir Ali Khan, Soumya Pati, Zulkifli Mustafa, Jafri Malin Abdullah, Hasnan Jaafar

**Affiliations:** Department of Pathology, School of Medical Sciences, Universiti Sains Malaysia, Jalan Hospital Universiti Sains Malaysia, 16150 Kubang Kerian, Kota Bharu, Kelantan Malaysia; Department of Applied Biology, College of Sciences, University of Sharjah, Emirates of Sharjah, 27272 United Arab Emirates; Department of Paediatric Neurology, Jan and Dan Duncan Neurological Research Institute at Texas Children’s Hospital, Baylor College of Medicine, Houston, TX USA; Director, Center for Neuroscience Services and Research, Universiti Sains Malaysia, Jalan Hospital Universiti Sains Malaysia, 16150 Kubang Kerian, Kota Bharu, Kelantan Malaysia; Department of Neurosciences, School of Medical Sciences, Universiti Sains Malaysia, Jalan Hospital Universiti Sains Malaysia, 16150 Kubang Kerian, Kota Bharu, Kelantan Malaysia; Jabatan Neurosains, Hospital Universiti Sains Malaysia, Jalan Hospital Universiti Sains Malaysia, 16150 Kubang Kerian, Kota Bharu, Kelantan Malaysia

**Keywords:** Mesenchymal stem cells, Neural progenitor-like cells, Insulin-like growth factor 1, Proliferation, Cell death

## Abstract

**Background:**

There has been increasing interest recently in the plasticity of mesenchymal stem cells (MSCs) and their potential to differentiate into neural lineages. To unravel the roles and effects of different growth factors in the differentiation of MSCs into neural lineages, we have differentiated MSCs into neural lineages using different combinations of growth factors. Based on previous studies of the roles of insulin-like growth factor 1 (IGF-1) in neural stem cell isolation in the laboratory, we hypothesized that IGF-1 can enhance proliferation and reduce apoptosis in neural progenitor-like cells (NPCs) during differentiation of MSCs into NCPs.

We induced MSCs differentiation under four different combinations of growth factors: (A) EGF + bFGF, (B) EGF + bFGF + IGF-1, (C) EGF + bFGF + LIF, (D) EGF + bFGF + BDNF, and (E) without growth factors, as a negative control. The neurospheres formed were characterized by immunofluorescence staining against nestin, and the expression was measured by flow cytometry. Cell proliferation and apoptosis were also studied by MTS and Annexin V assay, respectively, at three different time intervals (24 hr, 3 days, and 5 days). The neurospheres formed in the four groups were then terminally differentiated into neuron and glial cells.

**Results:**

The four derived NPCs showed a significantly higher expression of nestin than was shown by the negative control. Among the groups treated with growth factors, NPCs treated with IGF-1 showed the highest expression of nestin. Furthermore, NPCs derived using IGF-1 exhibited the highest cell proliferation and cell survival among the treated groups. The NPCs derived from IGF-1 treatment also resulted in a better yield after the terminal differentiation into neurons and glial cells than that of the other treated groups.

**Conclusions:**

Our results suggested that IGF-1 has a crucial role in the differentiation of MSCs into neuronal lineage by enhancing the proliferation and reducing the apoptosis in the NPCs. This information will be beneficial in the long run for improving both cell-based and cell-free therapy for neurodegenerative diseases.

## Background

Stem cells are generally defined as immature cells with self-renewal competencies and the capability of differentiating into other cell types [1, 2]. Stem cells may be differentiated into a wide range of mature cell types, and because of their differential potential they may be classified into two types: embryonic stem cells (ESCs) and adult stem cells (ASCs) [3]. ESCs are pluripotent cells derived from the inner cell mass of the blastocyst [4], whereas ASCs are isolated from adult tissues and defined as multipotent, since they are determined to produce cell types within their niche [5]. However, recent studies suggested that ASCs, especially mesenchymal stem cells (MSCs) derived from bone marrow (BM), have intrinsic neurogenic potential and may be differentiated into mature cells of ectodermal origin [6–8].

MSCs are also known as stromal cells and can be readily isolated from bone marrow and expended in vitro. In fact, MSCs have also been found in most postnatal organs such as the umbilical cord, adipose tissue, peripheral blood, and amniotic fluid [9–11]. The trans-lineage differentiation capability and unique immunogenic properties of MSCs have made them a potential alternative for cellular transplantation use in regenerative medicine [12]. Autologous transplantation of MSCs in stroke patients was proven therapeutically effective [13].

Recent studies have demonstrated that chemokines such as epidermal growth factor (EGF) and basic fibroblast growth factor (bFGF) are crucial factors in the neurogenic differentiation of MSCs [14, 15]. Our previous study has proved that a combination of EGF, FGF-2, and IGF-1 significantly enhances long-term proliferation and survival of rat striatal neural stem cells in vitro [16]. No analytical studies that we know of have been undertaken to investigate the effects of IGF-1 on neuronal induction of MSCs.

Therefore, the aim of this study was to analyse the role of IGF-1 in comparison with other types of growth factors that focus on stage-specific differentiation of MSCs into NPCs.

## Results

### Characterization of BM MSCs

**Figure 1:**
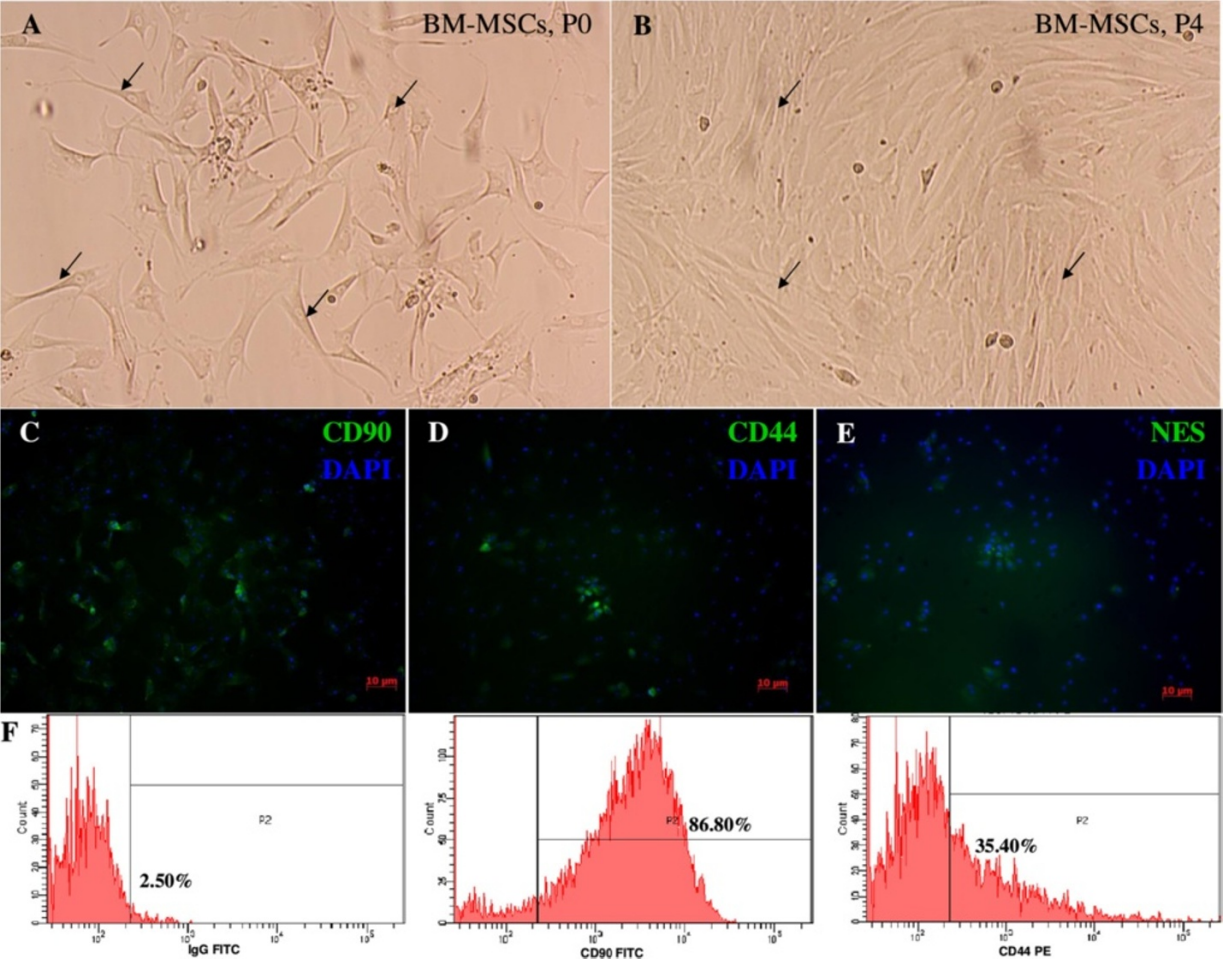
**Phenotypic analysis of bone marrow mesenchymal stem cells. (A)** Primary rat bone marrow MSCs attached to the surface of the flask exhibiting elongation (arrow). **(B)** Homogenous rat BM MSCs at passage 4 displaying a fibroblast-like appearance and spindle-like arrangement. **(C-D)** Immunofluorescence of BM MSCs showing a strong expression of CD90 **(C)** and CD44 **(D)**. **(E)** BM MSCs showing weak expression of nestin. **(F)** Immunophenotypes of BM MSCs analysed by flow cytometry. Images were viewed at 400× magnification using an inverted microscope and a fluorescence microscope equipped with a pseudo-fluorescence filter.

BM MSCs have fibroblast-like morphology (Figure 1A) and form whirlpool-like structures when grown to 80% confluence (Figure 1B). Immunofluorescence staining showed bright staining in all MSCs with antibodies against CD90 (Figure 1C) and CD44 (Figure 1D). Only 5.15 ± 0.68% (*n* = 6) of cells expressed nestin (Figure 1E). No staining was observed against markers of neuronal cell types such as Sox-2, GFAP, and FluoroPan (data not shown). Flow cytometry analysis further showed that BM MSCs were positive for CD90 and CD44 (Figure 1F). BM MSCs were subcultured until passage 4 and showed consistent morphology and phenotype. The phenotypic analyses of the cells isolated are similar to those described in Hong *et al*. [9], indicating that the BM MSCs isolated for our experiment met the standard criteria of mesenchymal stem cells.

### Differentiation of BM MSCs into NPCs

**Figure 2:**
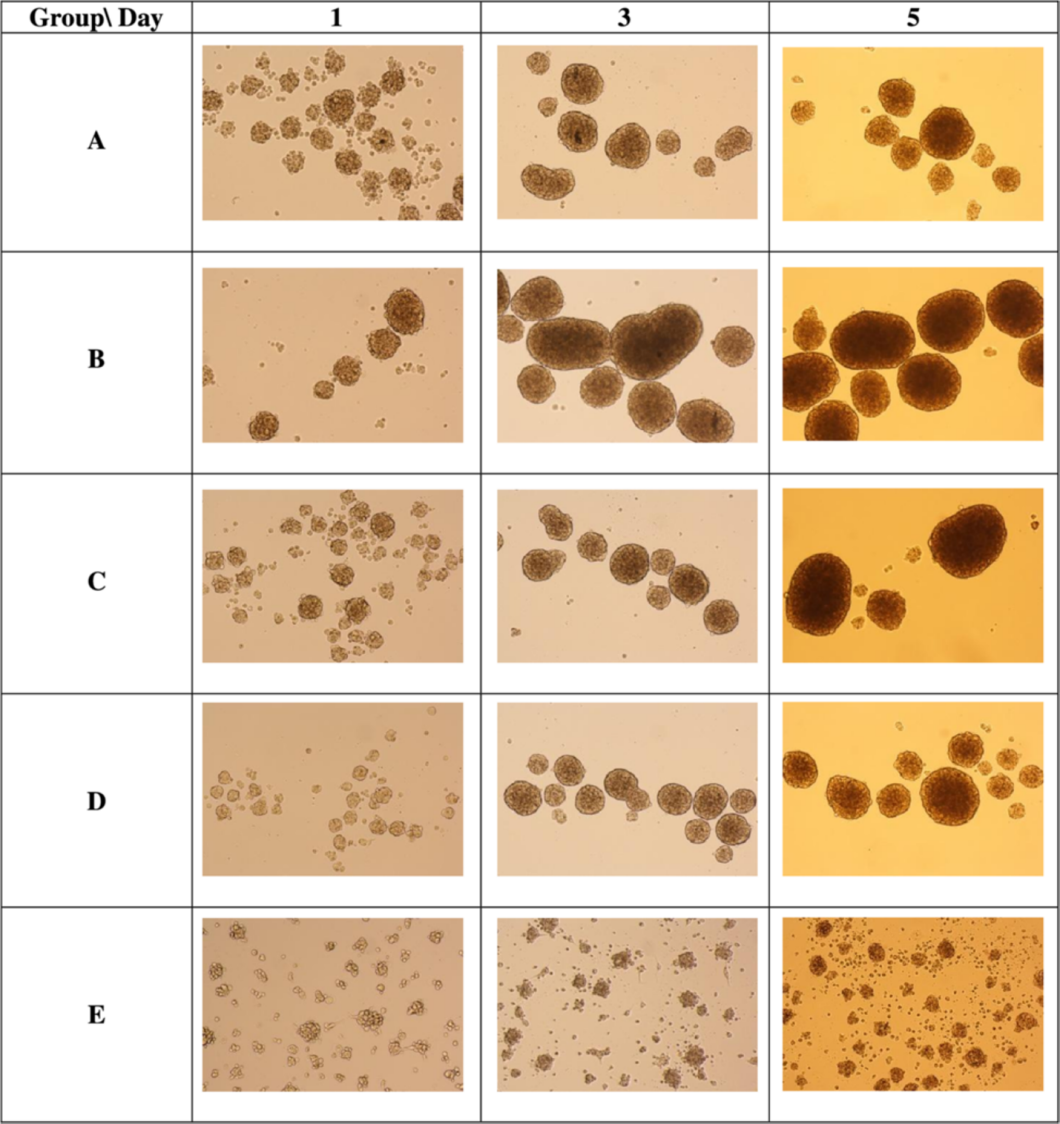
**Neurospheres at days 1, 3, and 5 showing differing sizes in different combination of growth factors.** Group **A** (EGF + bFGF); Group **B** (EGF + bFGF + IGF-1); Group **C** (EGF + bFGF + LIF); Group **D** (EGF + bFGF + BDNF) and Group **E** (no growth factors). Cells in Group **E** attached to the flask starting to differentiate (arrows). Neurospheres with irregular shape formed in Group E on day 5 of culture. Images were viewed with 100× magnification under an inverted microscope.

**Figure 3:**
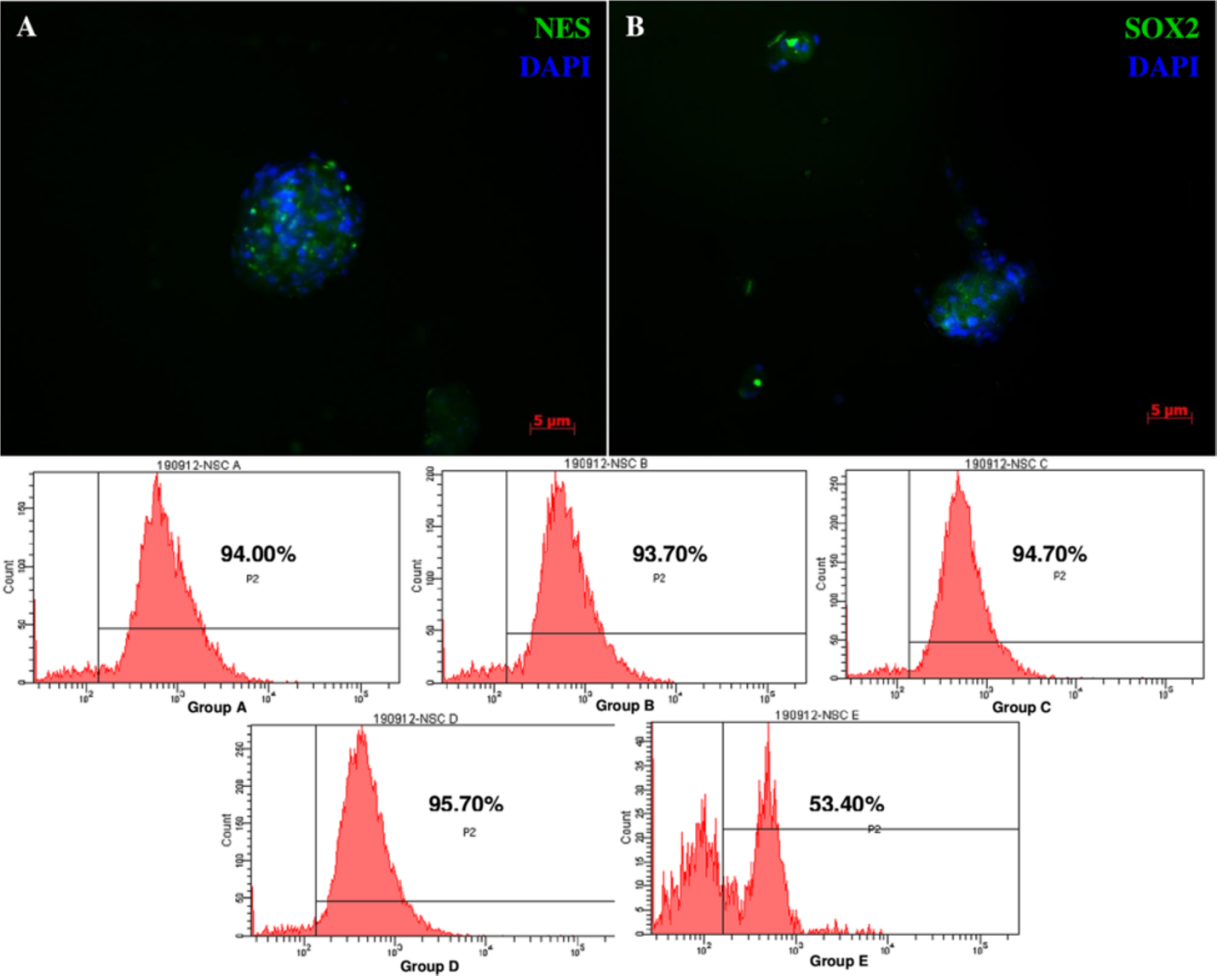
**Immunocytochemical staining and flow cytometer analysis of MSC-induced neural progenitor-like cells. (A)** NPCs from group B (EGF + bFGF + IGF-1) strongly expressing nestin. **(B)** NPCs from group B also strongly express Sox-2. **(C)** Flow cytometer analysis of nestin expression of NPCs from all groups **(A, B, C and D)** indicating the early differentiation of MSCs into NPCs. Group **E** clearly show the existence of two populations of cells indicating partial differentiation due to the effect of neural basal media. Flow cytometric data further validated the results obtained from immunofluorescence staining for group B. Images were viewed at 100× magnification using a fluorescence microscope.

Only the cells in Group E adhered to the surface of the tissue culture plates (Figure 2); the others formed many small spheres of floating cells after day 1 of induction. MSC-derived NPCs proliferated for 1 week without changing morphology or phenotype. Immunofluorescence staining showed that NPCs derived from group B (EGF + bFGF + IGF-1) were strongly positive for nestin (Figure 3A) and Sox-2 (Figure 3B). Moreover, data from flow cytometry confirmed the findings of immunocytochemical analysis (Figure 3C) and it also confirmed the expression of nestin by NPCs derived from all groups. In the 10,000 events acquired, 93.7% of the cells in Group B under IGF-1 treatment expressed nestin, whereas 53.4% of the cells in Group E expressed nestin and this expression might be due to the effect from neurobasal media. These results indicate the level of nestin-positive cells under the effect of the growth factors.

### Proliferation assays

**Figure 4:**
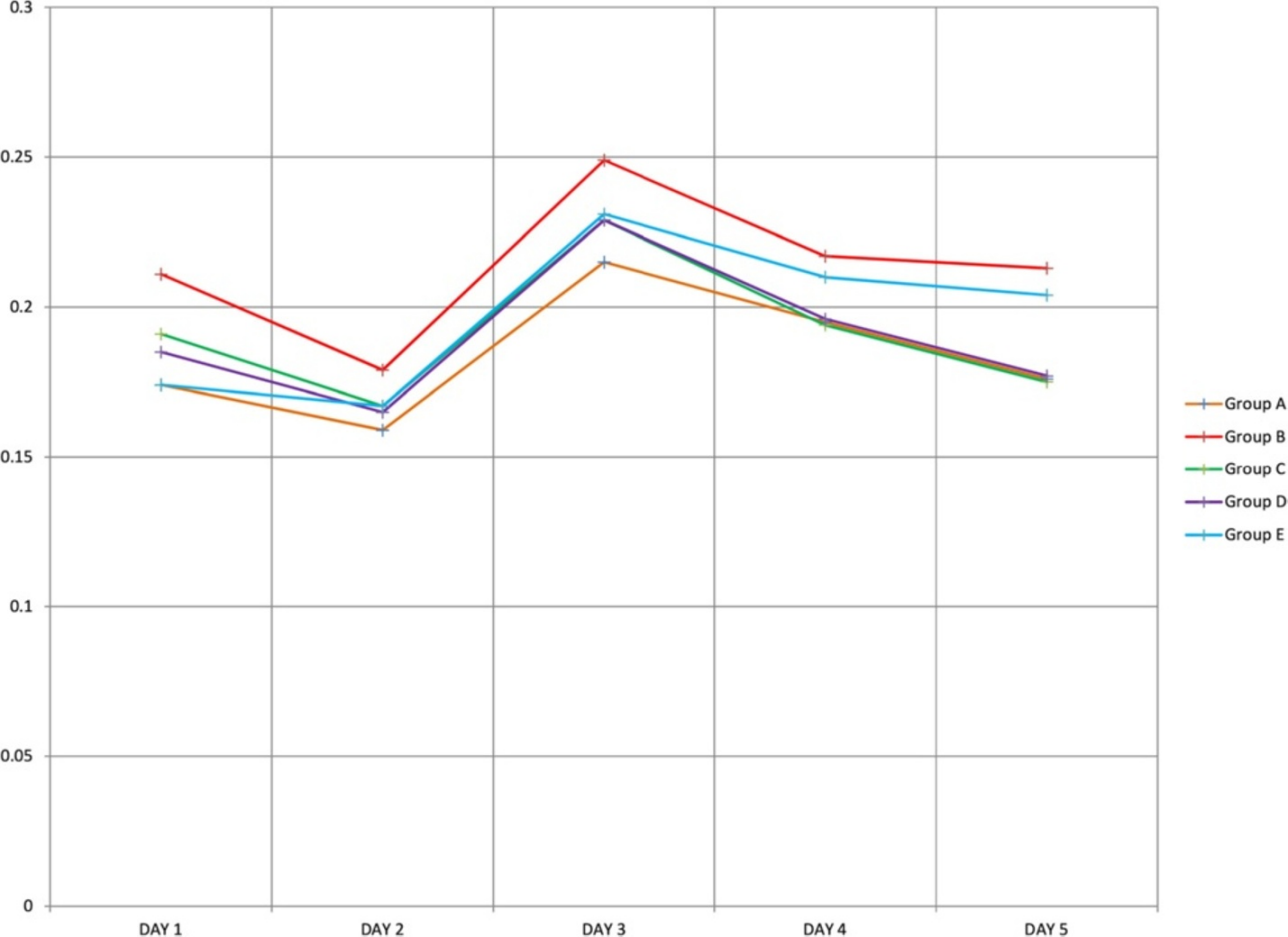
**Proliferation analyses of NPCs under different growth factors.** Cells (*n* = 12) were incubated with MTS reagent for 4 hr and changes in proliferation were studied at five time intervals (24 hr, 48 hr, 72 hr, 96 hr, and 120 hr). The data represented as optical density (OD) at 540 nm in mean ± SEM (*p* < 0.05).

**Figure 5:**
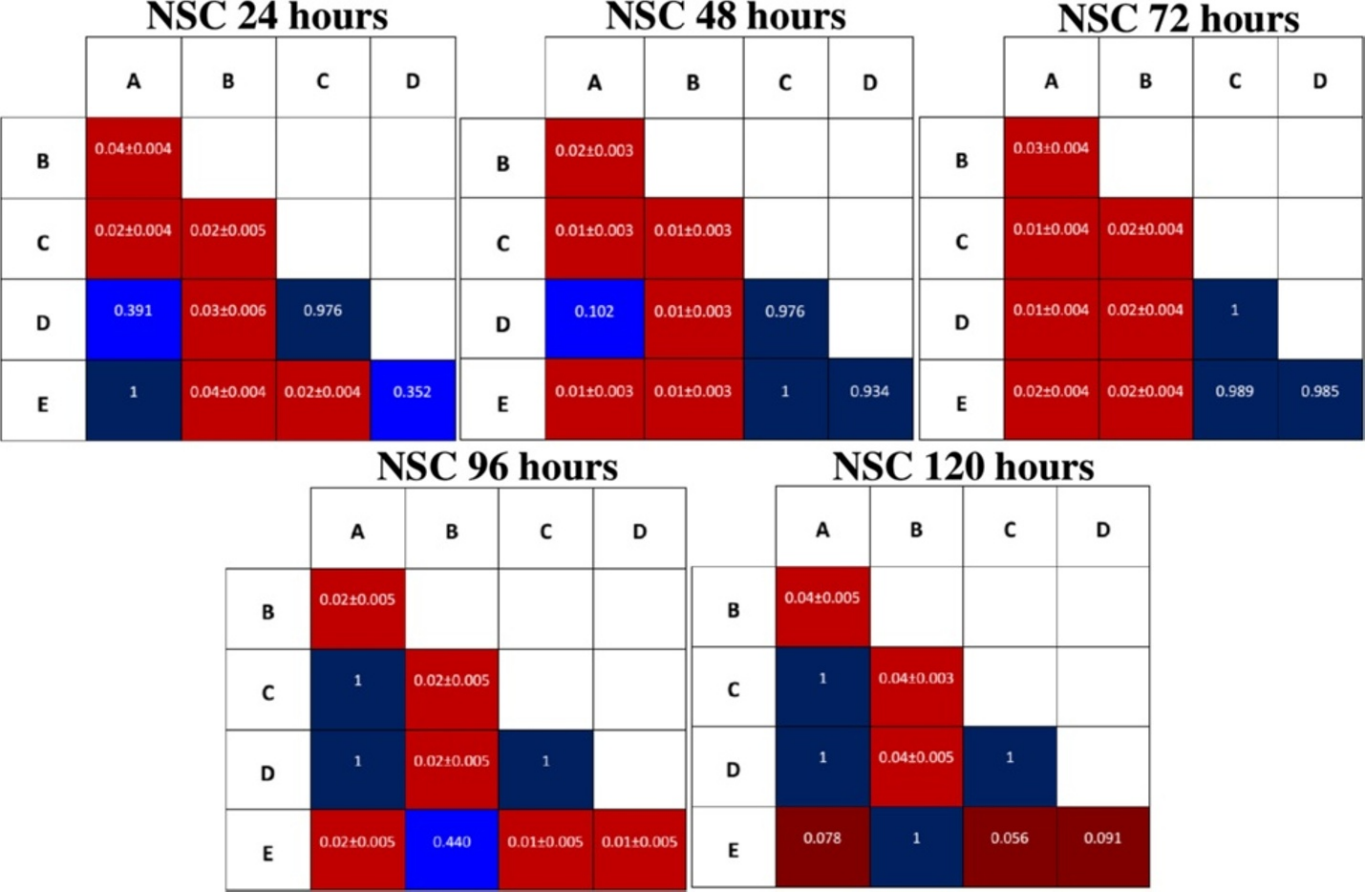
**Pairwise multiple comparison analyses between groups of NPCs at different time intervals.** Pairwise multiple comparison analysis was carried out using Tukey’s HSD (Tukey’s honest significant difference test). The data in red represents mean ± SD for statistically significant results (*p* < 0.05). *P*-*values* stated in blue indicate statistically insignificant comparisons (*p* > 0.05).

To elucidate the effects of growth factors on proliferation of NPCs we investigated the growth of cells using MTS assays at different time intervals (24 hr, 48 hr, 72 hr, 96 hr, and 120 hr) during the differentiation. Our results showed that the treatment group with IGF-1 had a significantly enhanced proliferation of NPCs at each time interval (Figure 4). To validate these data, we performed a one-way ANOVA across the groups. This demonstrated that IGF-1 could maintain cell proliferation at all time points (*n* = 12, *p* < 0.05). Our results were consistent with the previous study by Supeno *et al*. [16], which found that IGF-1 enhanced the proliferation of neural stem cells. We also carried out multiple comparisons between the groups (Figure 5). These comparisons clearly showed the effects of other growth factors in maintaining cell proliferation and their significance in differentiation activity. However, IGF-1 enhanced proliferation in NPCs more than the other growth factors.

### Apoptosis assay

**Figure 6:**
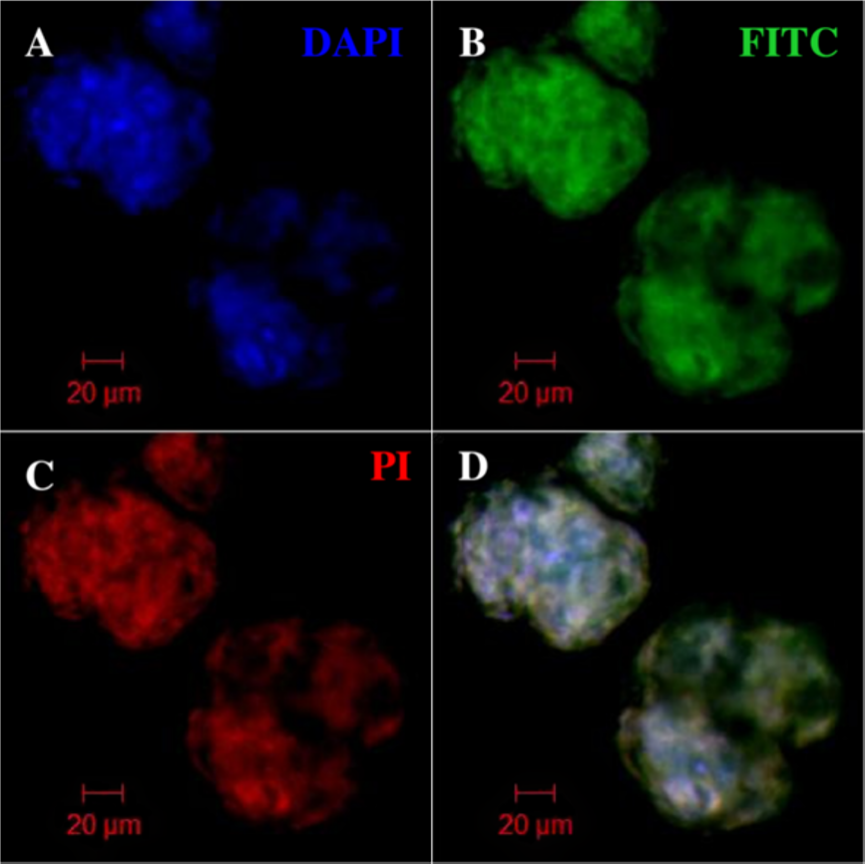
**Apoptosis assay.** Immunostaining revealed that transdifferentiation is associated with apoptotic activities. **(A)** SYTOX Blue nuclei staining, **(B)** FITC-conjugated Annexin V, **(C)** propidium iodide (PI), and **(D)** overlay image. All images are viewed at 40× magnification under a confocal microscope.

**Figure 7:**
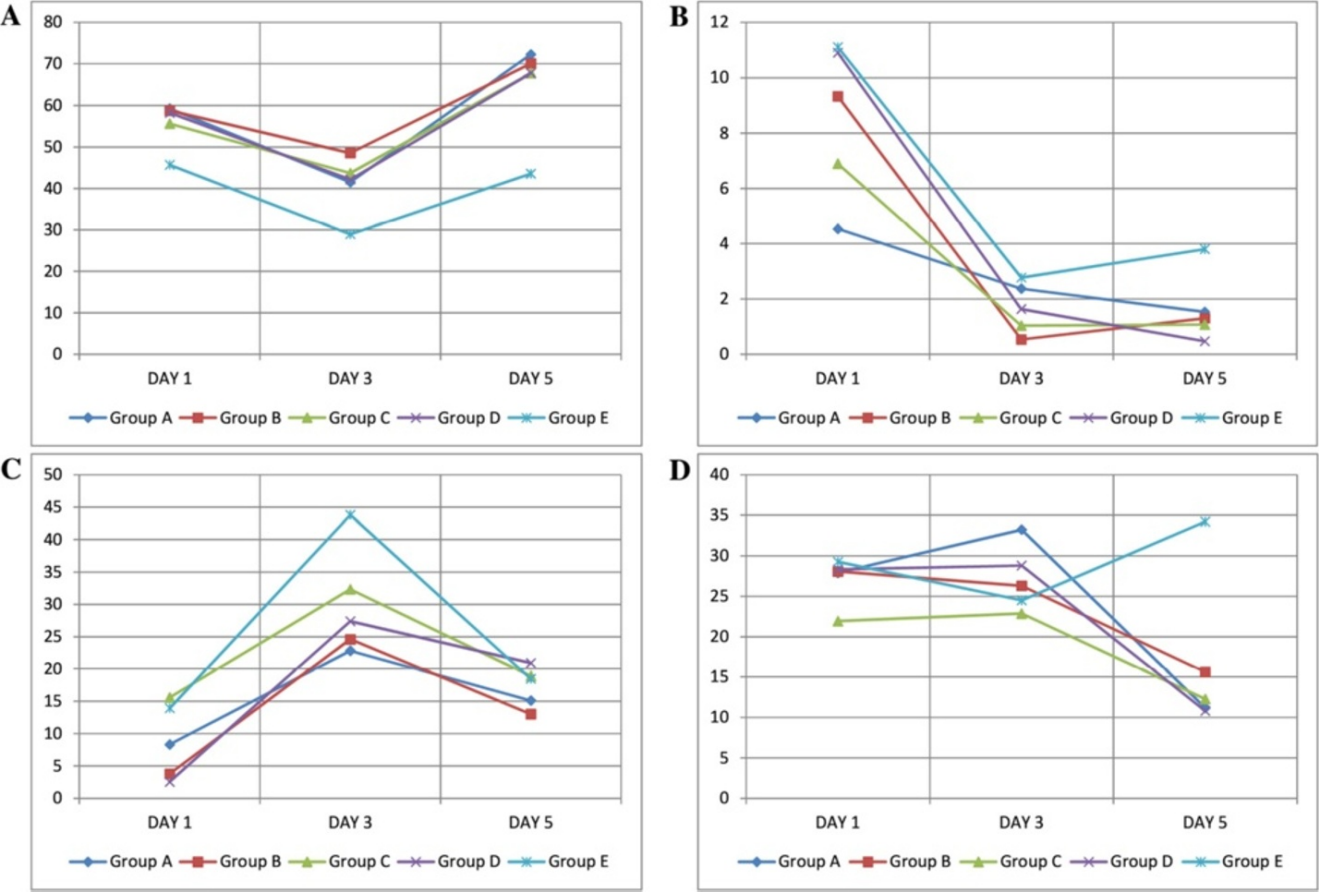
**Comparison of apoptotic activities between groups (** ***n*** **= 3). (A)** Percentage of live cells. **(B)** Percentage of necrotic cells. **(C)** Percentage of early apoptotic cells. **(D)** Percentage of late apoptotic cells. The data are presented as mean ± SD for statistically significant results (*p* < 0.05) and were obtained by flow cytometry equipped with analytical software.

To understand the role of the combination of growth factors on the survival of NPCs in vitro, we also studied the apoptotic activity of NPCs at three time intervals (24 hr, 72 hr, and 120 hr) using the Annexin V kit. Immunofluorescence staining of NPCs revealed that the differentiation process induces cellular apoptosis (Figure 6). Apoptotic activities such as necrosis and early and late apoptosis were quantified by flow cytometry. The group treated with IGF-1 maintained the highest percentage of live cells at day 3 post induction, 48.6 ± 19.5% (*n* = 3; Figure 7A). Only 9.3 ± 7.6%, 0.5 ± 0.7%, and 1.3 ± 1.4% (*n* = 3) of cells in Group B underwent necrosis at day 1, day 3 and day 5 respectively (Figure 7B). Furthermore, only 3.7 ± 3.3% of cells in Group B underwent early apoptosis. The number increases to 24.6 ± 15% on day 3 and decreases to 13 ± 11.5% on day 5 (Figure 7C). A total of 28.1 ± 15.05% of cells from Group B underwent late apoptosis, decreasing to 26.3 ± 14.3% on day 3 and further decreasing to 15.6 ± 6.7% on day 5 (Figure 7D). Taken together, the early and late apoptosis graphs suggest that the group treated with IGF-1 has better survival efficiency than the rest of the groups.

### Quantitative RT-PCR

**Figure 8:**
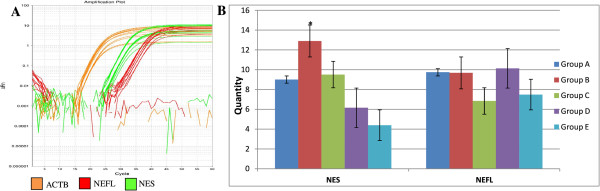
**Quantitative RT-PCR analysis (** ***n*** **= 3). (A)** Expression plots of the nestin gene (green), the neurofilament gene (red) and the housekeeping gene (orange). **(B)** Histograms showing quantification of NES and NEFL at the indicated time. Results are expressed as the mean ± SD. *p < 0.05.

Total RNAs were collected from each treatment group 3 days post induction, and real-time PCR validations on neurofilament gene (NEFL) and nestin (NES) were performed (Figure 8A). PCR reactions were normalised by amplifying the same sample of cDNA with primers against beta-Actin (ACTB). As expected, the treatment group with IGF-1 (Group B) expressed the highest level of nestin (*p* < 0.05, *n* = 3; Figure 8B), which was significantly greater than the other groups. However, the expression of the neurofilament gene did not show any significant variation in any of the treated groups. Our results suggest that IGF-1 maintains NPCs in an undifferentiated state while sustaining the stemness.

### Terminal differentiation of NPCs

**Figure 9:**
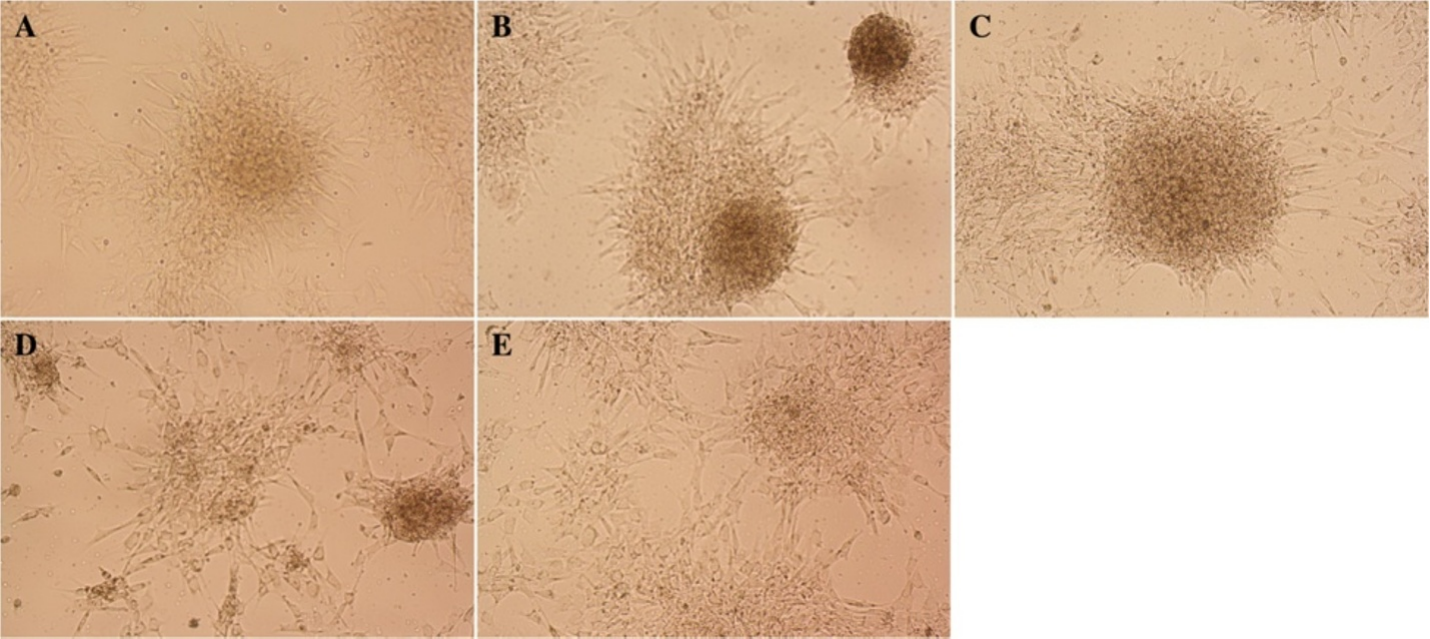
***In vitro*** **differentiation of NPCs into neuronal cell types.** NPCs from group **A**, **B**, **C** and **D** were differentiated at day 3 post induction using glial induction and neuronal induction protocol on poly-L-lysine coated wells for 14 days. All the NPCs from the four groups differentiated, attached to the surfaces and changed their morphology. The cells from negative control, Group **E**, were also treated similarly but the cells at the end of differentiation did not attach indicating no mature neural cells were formed.

**Figure 10:**
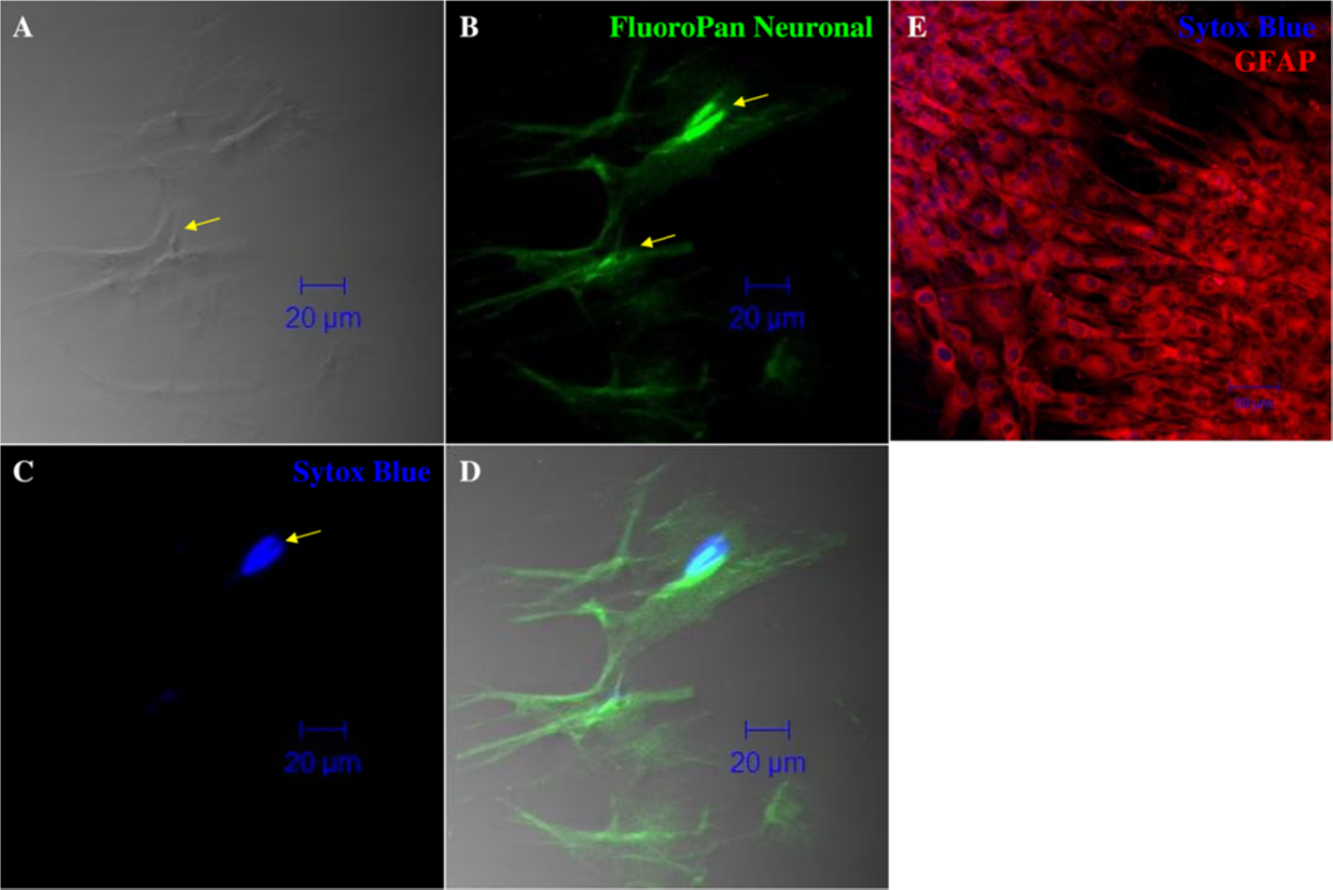
**Immunocytochemical staining of terminally differentiated cells.** NPCs from group B were differentiated using the neuronal induction medium and were stained with FluoroPan neuronal marker for neurons and astrocytes (GFAP). Nuclei have been counterstained with SYTOX Blue. **(A)** Phase contrast image of differentiated cells. **(B)** FluoroPan neuronal FITC. **(C)** Nucleus staining with SYTOX Blue. **(D)** Overlay image. **(E)** Astrocytes stained with GFAP and counterstained with secondary antibody Cy3. Images were taken at 400× magnification using a Pascal 5 confocal microscope (Carl Zeiss, Germany).

To assess the capability of NPCs to differentiate into mature neuronal cell types in vitro, we used the protocol suggested by Hermann *et al*. [14] with minor modifications. NPCs from groups A, B, C and D were plated in medium without EGF and bFGF but with brain-derived neurotrophic factor (BDNF) and platelet-derived growth factor (PDGF). All the NPCs in the four groups differentiated into mature neurons and glial cells. Cells attached and changed their morphology on the following day (Figure 9A-D). The cells from negative control, Group E, were also treated similarly but the cells at the end did not attach indicating no mature neural cells were formed (Figure 9E). The neural cells types formed most efficiently from the group B NPCs compared to other groups (Figure 9B). These differentiated cells, from group B NPCs, were further characterized with specific markers. The cells were allowed to differentiate for 10 days with constant replacement of growth factors. Differentiated cells were fixed in freshly prepared 4% PFA in PBS, blocked, and stained with FluoroPan neuronal marker and Glial fibrillary acidic protein (GFAP). We find it interesting that terminally differentiated NPCs expressed the marker specific for neuronal protein (Figures 10A-D) which commonly express in matured neuronal bodies and also GFAP (Figure 10E), an astrocytes marker. Moreover, differentiated cells also showed the characteristics of mature neuronal and glial cells. Thus our results have proved that NPCs are capable of being differentiated into neurons and astrocytes by specific cytokines supplementation. The results clearly indicate the NPCs derived from the group B were able to differentiate into mature neuronal phenotype.

## Discussion

Adult stem cells, particularly mesenchymal stem cells, have been used in neuro-regenerative medicine for the treatment of diseases such as stroke and traumatic brain injury [9]. The potential application of MSCs from bench to bedside is due to their easy accessibility and expansion capability both in vivo and in vitro, and also their ability to promote endogenous neurogenesis via a variety of secretion factors. Autologous transplantation of MSCs extracted from Wharton’s jelly has been reported to be free from immunogenic rejection [18]. It has been reported that MSCs can be induced into neural lineage through several methods such as incubation with cytokines [19], co-culture with nerve cells [1, 6], and transfection with known factors [20]. Recent evidence has shown that synergistic treatment of MSCs with bFGF and EGF significantly differentiates MSCs into cells expressing neural lineage markers. Previous studies in the laboratory have shown that addition of IGF-1 leads to enhanced embryonic striatal neural stem cell proliferation in vitro [16]. In the present study, we have demonstrated that the presence of IGF-1 enhanced proliferation and reduced apoptosis during the differentiation of MSCs into NPCs.

Nestin, the intermediate filament protein, is predominantly expressed during early neurogenesis and also expressed in neural stem cells [7]. Immunofluorescence staining of MSCs showed mild expression of nestin (Figure 1E) consistent with the previous finding of Wislet-Gendebien *et al*. [1] that nestin-positive MSCs may differentiate in vitro into functional neuronal-like cells. From recent studies it can be suggested that MSCs have two populations: nestin positive and nestin negative [1]. Some research suggests that the MSCs express the nestin when they are under stress [21, 22]. However, further research is warranted to characterise the two populations of the MSCs and trace their origins. In our study, neurosphere formation was observed in all groups except Group E, and the size of the neurospheres increased every day (Figure 2), indicating that cell proliferation occurs during the differentiation. The phenotype and morphology of MSC-derived neurospheres in our study are similar to that of embryonic striatal stem cells isolated previously in the laboratory [16]. During the differentiation, nestin expression increases sharply (Figure 3C), and immunochemical staining showed that almost all the cells were positive for nestin and Sox-2 after 5 days. This indicated that the MSCs have switched from their original mesodermal lineage into ectodermal and started differentiate into more lineage specific cells such as neurons or astrocytes. Flow cytometry analysis of Group E (Figure 3C) clearly showed that without growth factors a few MSCs were differentiated into NPCs. This differentiation may be due to either the effect of neural basal media or in vitro stress. The nestin expression was highest in the NPCs derived from IGF-1, indicating that the growth factor enhances the proliferation of NPCs during the differentiation of MSCs into NPCs. We investigated the proliferation activity of each group due to the effect of the respective growth factors. Our result strongly corroborated with the previous finding that IGF-1 treatment could significantly enhance proliferation of NPCs in vitro (Figure 4). Since our aim was to study the early stages of neuronal differentiation, a proliferation assay was carried out in a stage-specific approach on passage 1 at five different time intervals. We hypothesized that the cells would undergo apoptosis at day 2 and start to stabilise at day 3 post-induction. This is consistent with the previous finding that one third of MSCs died after induction [14]. IGF-1 has mitogenic effects by promoting G_1_/S cell cycle progression through Akt-dependent and tyrosine kinase–signalling pathways [23–25]. These signalling pathways activate cell cycles and anti-apoptotic pathways; hence NPCs formed will proliferate efficiently in the presence of IGF-1 [22]. We also carried out intergroup comparisons, and the data was presented in checkbox format (Figure 5). Our data has clearly indicated that IGF-1 has positive regulatory effects in maintaining cellular propagation of NPCs in neuronal differentiation.

MSCs may even release certain neurotrophic factors such as BDNF, nerve growth factor, and glial-derived neurotrophic factors [26, 27], and it is well known that the neurotrophic factors are important for maintaining cellular survival of neuronal cells both in vitro and in vivo. Immunochemistry showed that neurospheres from all groups undergo qualitatively similar apoptosis (Figure 6). Therefore, we proceeded to quantitative analysis using flow cytometry, and the data were presented in four different graphs, which indicated the percentages of live cells, necrotic cells, and early and late apoptotic cells (Figure 7). Cells treated with IGF-1 showed generally better cell survival, maintaining a higher percentage of live cells and a lower percentage of both necrotic and apoptotic cells during differentiation. Moreover, our results also suggested that since MSCs could self-secrete BDNF, which is crucial for neuroprotection, and our findings show that IGF-1 promotes better survivability than those induced under BDNF influence alone (Group D). We suggest that IGF-1 might interact with BDNF to promote better enhancement of terminal differentiation.

Results of both proliferation and apoptosis have led us to conclude that NPCs achieve optimal condition 3 days post induction. Thus we investigated the quantity of nestin and neurofilament genes expressed at day 3 from all groups. Our results showed that the group treated with IGF-1 expressed the highest nestin level of all groups. This indicated that IGF-1 effectively maintains the stemness of the neural progenitor cells and the combination of the growth factors maintains the cells in an undifferentiated stage; our findings support those in the previous study by Chang *et al*. [28].

Furthermore, we successfully differentiated neurospheres from all groups into cells that expressed neuronal markers and astrocytes (GFAP; Figures 9 and 10). Thus, our neurospheres induced from MSCs are self-proliferating cells that maintain the capacity to differentiate into mature brain cells.

We differentiated neurospheres from each group according to Hermann *et al*. [14]. Neurospheres from all groups terminally differentiated into cells with a neuronal phenotype. The neural phenotype (mature brain cells) formed from neurospheres treated with IGF-1 are shown in the Figure 9. The terminal differentiation of neurospheres in the group B was most efficient compared to other three groups. Thus our results showed clearly that the combination of growth factors EGF + FGF-2 + IGF-1 enhances proliferation and reduces the apoptosis in the NPCs during the differentiation of MSCs into neural lineages. The IGF-1 treatment produced healthier and more functional neural progenitor-like cells compared to other combinations of growth factors tested.

## Conclusion

We conclude that IGF-1 is an important growth factor for enhancing proliferation and inhibiting apoptosis of neural progenitor-like cells during differentiation of MSCs into neural lineages. Further, IGF-1 enhances nestin expression. These enhancements might be due to the activation of tyrosine kinase– and PI3 kinase–signalling pathways, which are mediated by the IGF-1 growth factor [23–25]. These pathways activate proliferation and inhibit apoptosis in the NPCs. This information will be beneficial for future improvements in both cell-based and cell-free therapy for neurodegenerative diseases.

## Methods

### Animals

Sprague Dawley (SD) rats 4–6 weeks old were obtained from the Animal Research and Service Centre (ARASC, Universiti Sains Malaysia, Health Campus). Housing and breeding of the rats were taken care of in intramural facilities. The animal ethics clearance for all experimentation was obtained from the USM Animal Ethics Committee.

### BM MSC cultures

The SD rats were euthanized by intra-peritoneal injection of an overdose of ketamine (100 mg/ml) and xylazine (100 mg/ml) from Ilium Troy Laboratory, Australia. Femoral and tibiae bones were then dissected aseptically. The central canal of the bone was injected with 5 ml of Dulbecco’s Modified Eagle’s Medium (DMEM, catalog # 11885–084, Gibco, Life Technologies, USA) containing 20% (v/v) fetal bovine serum (FBS, catalog # 10270098, Gibco, Life Technologies, USA), 1% MEM Non-essential amino acid (Catalog # M7145, Sigma-Aldrich, USA) and 1% Antibiotic-antimycotic (Catalog # 15240062, Gibco, Life Technologies, USA) to extrude the marrow tissue. Then the tissue was gently dissociated into single cells mixture and layered onto a Ficoll-Paque PREMIUM gradient solution (Catalog # 17544202, GE Healthcare Bio-sciences, Sweden) and centrifuged at 2000 rpm for 20 min. Mononuclear cells were extracted and plated on T25 cm^2^ tissue culture flasks at a density of 1 × 10^7^ marrow cells and incubated in a humidified chamber at 37°C with a 5% CO_2_ supply. After 24 hours the non-adherent cells were removed by total media replacement, and the attached cells were grown. When the MSCs became 80% confluent, they were detached with 0.25% trypsin/EDTA and then subcultured. BM MSCs were characterised by flow cytometry, and neuronal induction was conducted at passage 4.

### In vitro differentiation of BM MSCs into neural-like cells

The differentiation protocols used in this study were modified from Supeno *et al*. [16]. BM MSCs were dissociated with 0.25% trypsin-EDTA and reseeded into an Ultra-Low Attachment 24-wells plate (Catalog # C62-3473, Corning, USA) at a concentration of 1 × 10^5^ cells/cm^2^ in NeuroCult® NS-A proliferation media specific for rat (Catalog # 05771, STEMCELL Technologies, Canada). The cells were then supplemented with four different combinations of growth factors. In accordance with the study design, the BM MSCs were cultured in five experimental groups: Group A (EGF + bFGF), Group B (EGF + bFGF + IGF-1), Group C (EGF + bFGF + LIF), Group D (EGF + bFGF + BDNF), and the negative control, Group E (no growth factor). All groups of cells were maintained in the incubator at 37°C with a 5% CO_2_ supply. On the second and fourth day of cell culture, 50% of the media was replaced and fresh growth factors were added. Rate of cell proliferation and cell death of the neurospheres formed were analysed after three time intervals (24 hr, 3 days, and 5 days) of *in vitro* culturing. Induction of terminal neural differentiation was then initiated according to Hermann *et al*. [14], with minor modification. The neurospheres were plated on glass slides coated with poly-D-lysine, 10–20 neurospheres per ml of NeuroCult® supplemented with 10 ng/ml PDGF-BB recombinant mouse protein (Catalog # PMG0043, Invitrogen, USA) for glial induction or 10 ng/ml rh-BDNF (Catalog # GF029, Chemicon International, Millipore, USA) for neuronal differentiation. Cells were differentiated for 14 days and media was changed every week with growth factors supplementation every 2 days.

### Immunocytochemistry characterization

The BM MSC and neurosphere phenotypes were determined by immunological labelling. Cells were fixed in 4% paraformaldehyde in PBS for 10 min at room temperature and blocked with a solution containing 5% of bovine serum albumin in PBS and 0.05% Triton X-100 for cell permeabilization. Immunocytochemistry was carried out according to standard protocols. Cell nuclei were counterstained with SYTOX® Blue nucleic acid stain (1:1000; Invitrogen, USA). The antibodies used and dilution were as follows: Anti-CD90 (FITC) conjugated mouse monoclonal antibody (1:200; Thermo Scientific, USA); Anti-CD44 (PE) conjugated rat monoclonal antibody (1:200; Abcam, Cambridge, UK) [17]; Alexa Fluor® 647 mouse anti-nestin (1:200; BD Pharmingen, USA); Milli-Mark™ FluoroPan neuronal marker (1:100; Catalog # MAB2300X, Millipore, USA); Anti-Sox-2 clone 6G1.2 FITC conjugated antibody (1:100; Millipore, USA); Rabbit anti-GFAP (1:100; Millipore, USA) and donkey anti-rabbit IgG-FITC secondary antibody (1:250; Santa Cruz Biotechnology, Inc, USA). UltraCruz™ anti-fading mounting medium (Catalog # Sc-24941, Santa Cruz Biotechnology, Inc, USA) was applied to minimise photo bleaching due to exposure to high-intensity light. The fixed cells were then viewed using a Pascal 5 confocal microscope (Carl Zeiss, Germany).

### Flow cytometric analysis

Nestin expression of MSC-derived NPCs was quantified by flow cytometry analysis. Neurospheres at different time intervals were dissociated into single cells using a solution of Detachin (Catalog # T100100, Genlantis, USA). Next, the cells were permeabilized and incubated with Alexa Fluor® 647 mouse antibody against nestin on ice for 1 hr. A FACSCanto flow cytometry machine equipped with FACSDiva analytical software (BD Bioscience, USA) was used for analysing the percentage of nestin-positive cells. A minimum of 10,000 events was acquired for each sample. Forward and side light-scatter gates were set to exclude debris and clumps of cells.

### Proliferation study

Proliferation studies were performed using CellTiter 96® Aqueous One Solution cell proliferation assay (Catalog # G3580, Promega, USA). BM MSCs were seeded in 96-wells cell culture plates (1000 cells/well) within the respective neuronal induction media (Group A: EGF + FGF-2; Group B: EGF + FGF-2 + IGF-1; Group C: EGF + FGF-2 + LIF; Group D: EGF + FGF-2 + BDNF; and Group E: without growth factor). On the first, third, and fifth days of culture, cells were incubated with 3-(4,5-dimethylthiazol-2-yl)-5-(3-carboxymethoxyphenyl)-2-(4-sulfophenyl)-2H-tetrazolium (MTS) reagent at 37°C for 4 hours, and absorbance was measured using a spectrophotometer at wavelength 490 nm.

### Apoptosis assay

Apoptotic activity due to neuronal induction was assessed using Annexin V stain (Catalog # 556547, BD Pharmingen, USA). Neurospheres from each group were gently dissociated into single-cell format, resuspended in 1X binding buffer, and then incubated with FITC-conjugated Annexin V and propidium iodide for 15 minutes, according to the manufacturer’s recommended protocol. Apoptotic activity was observed under a confocal microscope, and quantitative results were obtained by flow cytometry analysis.

### RNA extraction and quantitative real-time PCR analysis

Total cellular RNAs were extracted from neurospheres of each group using the miRNeasy Mini Kit (Catalog # 217004, Qiagen, Germany). Next, reverse transcription was performed on up to 2ug of the total RNA using the High Capacity RNA-to-cDNA Kit (Catalog # 4387406, Applied Biosystems, USA) as directed by the manufacturer. The expressions of nestin and neurofilament genes were determined by the TaqMan® Real-Time PCR system (Applied Biosystems, USA). Predesigned primer and amplicons length were as follows: nestin (NES): Rn00564394m1, 78; neurofilament (NEFL): Rn00582365m1, 65; and the housekeeping gene beta-Actin (ACTB): Rn00667869m1, 91. Real-time quantitative analysis was carried out using the 7500 Real-Time PCR system (Applied Biosystems, USA) and amplification was monitored and analysed by 7500 software version 2.0.6 (Applied Biosystems, USA).

### Statistical analysis

Statistical analysis was carried out using IBM SPSS Statistics Software version 20. Results were analysed for statistical significance using a two-way ANOVA for different time intervals: day 1, day 3, and day 5. All data unless specified are expressed as the mean plus or minus the standard deviation. Values of *p* less than 0.05 were considered statistically significant.

## Abbreviations

BM-MSCs: Bone marrow mesenchymal stem cells
IGF-1: Insulin-like growth factor
LIF: Leukemia inhibitory factor
EGF: Epidermal growth factor
bFGF: Basic fibroblast growth factor
BDNF: Brain-derived neurotrophic factor
PDGF: Platelet-derived growth factor
NPCs: Neural progenitor-like cells
NES: Nestin
NEFL: Neurofilament
ACTB: Beta-actin
PFA: Paraformaldehyde
DMEM: Dulbecco’s modified Eagle’s media
PBS: Phosphate buffer saline
MTS: 3-(4,5-dimethylthiazol-2-yl)-5-(3-carboxymethoxyphenyl)-2-(4-sulfophenyl)-2H-tetrazolium
ASCs: Adult stem cells
ESCs: Embryonic stem cells
SD: Sprague Dawley
FITC: Fluorescein isothiocyanate
PE: Phycoerythrin
GFAP: Glial fibrillary acidic protein.
